# Gut microbiota, inflammatory factors, and scoliosis: A Mendelian randomization study

**DOI:** 10.1097/MD.0000000000038561

**Published:** 2024-06-14

**Authors:** Xiaojiang Zhao, Jingjing Liu, Lei Zhang, Chao Ma, Yanan Liu, Hebao Wen, Chang qing Li

**Affiliations:** a Department of Physical Education and Arts, Bengbu Medical College, Bengbu, China; b Graduate School, Adamson University, Manila, Philippines; c Physical Education Department, Bozhou University, Bozhou, China.

**Keywords:** gut microbiota, inflammatory factors, Mendelian randomization, scoliosis

## Abstract

Several studies have reported a potential association between the gut microbiota (GM) and scoliosis. However, the causal relationship between GM and scoliosis and the role of inflammatory factors (IFs) as mediators remain unclear. This study aimed to analyze the relationship between GM, IFs, and scoliosis. We investigated whether IFs act as mediators in pathways from the GM to scoliosis. Additionally, using reverse Mendelian randomization (MR) analysis, we further investigated the potential impact of genetic predisposition to scoliosis on the GM and IFs. In this study, we searched for publicly available genome-wide association study aggregate data and utilized the MR method to establish bidirectional causal relationships among 211 GM taxa, 91 IFs, and scoliosis. To ensure the reliability of our research findings, we employed 5 MR methods, with the inverse variance weighting approach serving as the primary statistical method, and assessed the robustness of the results through various sensitivity analyses. Additionally, we investigated whether IFs mediate pathways from GM to scoliosis. Three negative causal correlations were observed between the genetic predisposition to GM and scoliosis. Additionally, both positive and negative correlations were found between IFs and scoliosis, with 3 positive and 3 negative correlations observed. IFs do not appear to act as mediators in the pathway from GM to scoliosis. In conclusion, this study demonstrated a causal association between the GM, IFs, and scoliosis, indicating that IFs are not mediators in the pathway from the GM to scoliosis. These findings offer new insights into prevention and treatment strategies for scoliosis.

## 1. Introduction

Scoliosis is a prevalent musculoskeletal disorder affecting the spine, encompassing various spinal, thoracic, and trunk abnormalities. It is characterized by structural and alignment changes due to compositional alterations in the spine.^[1,2]^ The Scoliosis Research Society recommends a definitive diagnosis when Cobb angle measures 10° or greater and axial rotation is present.^[3]^ Severe scoliosis can induce chest and rib dysplasia and exacerbate spinal cord compression, leading to paralysis.^[4]^ Although the exact pathophysiological mechanism of scoliosis remains uncertain, prior research suggests that its development significantly involves the gut microbiota (GM) and immune system.^[2,5,6]^

The GM consists of diverse microbial populations in the intestinal tract that engage in symbiosis with the host organism, including bacteria and protozoa. This complex ecosystem can influence various physiological functions, including metabolism, inflammation, and immune responses.^[7,8]^ These gastrointestinal microbiota are also integral to maintaining physiological equilibrium and metabolic processes, contributing to immune system maturation, vitamin synthesis, and nutrient assimilation.^[9]^ Some studies have indicated that a dysregulation of the GM could disrupt hormonal balance and lead to metabolic disorders.^[10,11]^ Additionally, metabolic disorders and hormonal changes have been observed in adolescent idiopathic scoliosis and individuals with low bone density before the onset of scoliosis.^[12,13]^ Modifications in microbiome composition and host reactions to microbiota have been associated with abnormal bone growth and resorption,^[14]^ leading to the concepts of the “gut-musculoskeletal axis” and the “gut-bone axis.”^[15,16]^ Inflammation has also been identified as a potential risk factor for scoliosis.^[17]^ Epidemiological research has shown a correlation between elevated levels of general inflammation and a high prevalence of scoliosis. Moreover, the GM and IFs appear to influence the progression of scoliosis. Hence, we hypothesized that IFs mediate the pathway from GM to scoliosis.

Notably, current studies examining the relationship between GM or IFs and scoliosis are observational in nature, making them susceptible to confounding, reverse causality, and bias.^[18]^ While randomized controlled trials (RCTs) can assist in establishing a causal relationship between GM or IFs and scoliosis, conducting such trials in humans is not feasible owing to ethical considerations, subject compliance, and other constraints.^[19]^ MR analysis has emerged as a widely used method to complement RCTs, leveraging epidemiological data to draw conclusions about causality. MR studies consider genetic variations as instrumental variables (IVs), which are used to assess the causality between exposure and outcomes.^[20–22]^ Because genetic variation is established at conception, MR analysis is less susceptible to bias from environmental confounders and reverse causality than observational studies.^[22]^ In MR analyses, IVs can be drawn from the aggregated statistics of large, nonoverlapping genome-wide association studies (GWAS). Specifically, summary statistics can be used to examine the relationship between GM, IFs, and scoliosis.

In this study, we utilized the MR method to analyze the relationship between GM, IFs, and scoliosis. We explored whether IFs act as mediators in the pathway from the GM to scoliosis. Furthermore, through reverse MR analysis, we further investigated the impact of scoliosis on GM and IFs.

## 2. Materials and methods

### 2.1. Study design

**Figure 1.**
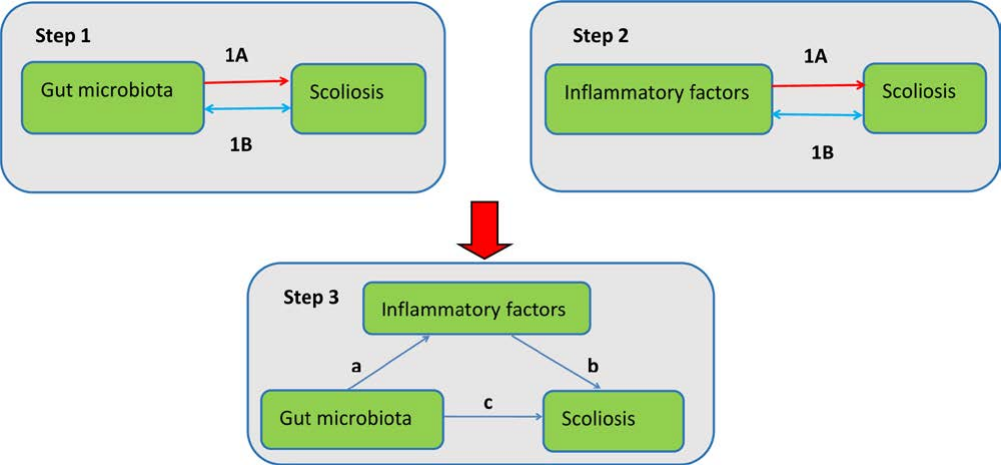
Study design. Step 1 – phase 1A represents the causal effect of GM on scoliosis. Step 1 – phase 1B indicates a bidirectional causal relationship between GM and scoliosis. Step 1 – phase 2A represents the causal effect of IFs on scoliosis. Step 1 – phase 2A represents the bidirectional causal effect between IFs and scoliosis. Step 2 represents the mediation analysis of IFs in the pathway between GM and scoliosis. GM = gut microbiota, IFs = inflammatory factors.

This study comprised 2 phases of analysis (Fig. 1). The first phase consisted of 2 parts. In the first part, we analyzed the relationship between GM and scoliosis using a 2-sample MR method. In the second part, we analyzed the relationship between IFs and scoliosis using 2-sample MR methods. The second phase involved analyses of the IFs mediating the pathway from GM to scoliosis.

MR analysis must satisfy 3 core assumptions.^[23]^ First is the assumption of relevance, where the IVs must be reliably associated with the exposure factor under study. Second is the independence assumption, where the IVs must not be related to known or unknown confounders. Third is the exclusion limitation assumption, where IVs must exclusively affect the outcome through the exposure factor and not through other direct causal paths.

### 2.2. Data sources

Pooled statistical data on gut microbes from the MiBioGen Consortium (https://mibiogen.gcc.rug.nl) were utilized in a large-scale GWAS involving 18,340 individuals of European minority descent from 11 countries, examining 122,110 mutation loci.^[7]^ The pooled GWAS data included information on 211 taxonomic groups of the GM. Genetic data for IFs were obtained from a prior GWAS involving 14,824 European participants and included information on 91 IFs.^[24]^

GWAS data on scoliosis were obtained from the FinnGen Consortium R10, published in December 2023, to match the ethnicity of the study participants (https://www.finngen.fi/en/access_results). The scoliosis GWAS from FinnGen, which had a sample size of 165,850 individuals, included 16,380,270 single nucleotide polymorphisms (SNPs) available for analysis after controlling for variables such as age, sex, and genotyping batch.^[25]^

### 2.3. Selection of IVs

Initially, a genome-wide significance threshold of *P* < 5 × 10^−8^ was established to identify SNPs that showed a strong correlation with GM and IFs. However, as only a few SNPs met this stringent criterion, a higher cutoff was chosen. Ultimately, we applied *P* < 1 × 10^−5^ and *P* < 5 × 10^−6^ as the significance thresholds for screening the IVs associated with GM and IFs, respectively. The threshold of significance for selecting IVs associated with scoliosis was *P* < 5 × 10^−8^. Additional criteria were implemented to enhance the reliability and precision of the outcomes when selecting the IVs. These criteria included checking for linkage disequilibrium (*r*^2^ = 0.001, kb = 10,000) to adhere to the assumptions of MR^[26]^ and excluding palindromic SNPs to mitigate the potential impact of alleles on causality results. The robustness of the genetic IVs was verified by calculating the *F* statistic using the formula *F* = (*n* − *k* − 1)/*k* × (*R*^2^/1 − *R*^2^).^[27]^ An *F* statistic above 10 indicates a robust IV.^[28]^

### 2.4. Statistical and sensitivity analyses

We employed bidirectional MR analyses to establish a causal relationship between the GM and IFs related to scoliosis. Five different methods were employed for this purpose: MR-Egger,^[29]^ weighted median,^[30]^ inverse variance weighted IVW,^[31]^ simple mode, and weighted mode, with particular emphasis on the results from the IVW method. If the *P* value of the IVW was <.05 and the *P* values of the other 4 methods were >.05, a causal relationship could still be inferred. However, the direction of the effect value (i.e., positive or negative) of the other 4 methods should be consistent with that of the IVW method.

Several methods were employed to ensure the robustness and reliability of our study. To detect potential heterogeneity, we used IVW, MR-Egger, and visualized funnel plots with a significance threshold of *P* < .05. Furthermore, we employed the MR-Egger regression intercept, MR-PRESSO global test, and scatter plots to assess horizontal pleiotropy,^[32]^ with a significance threshold of *P* < .05. A leave-one-out technique was also used to evaluate the robustness of our findings.

The GM and IFs, which exhibited significant causal relationships with scoliosis, were included in the mediation analyses using MR (step 1 – phase 1A and step 1 – phase 2B in Fig. 1). We also assessed whether GM had a causal relationship with IFs (step 2 in Fig. 1). If causality was established, we performed multivariate MR (MVMR) analyses to demonstrate whether IFs mediate the pathway from GM to scoliosis.

All analyses were performed using R software (version 4.3.2). MR analysis was performed using the R-based package “TwoSampleMR” and the “MR_PRESSO” package was used to conduct multiplicity tests.

## 3. Results

### 3.1. Causal effects of the GM and IFs on scoliosis

**Figure 2.**
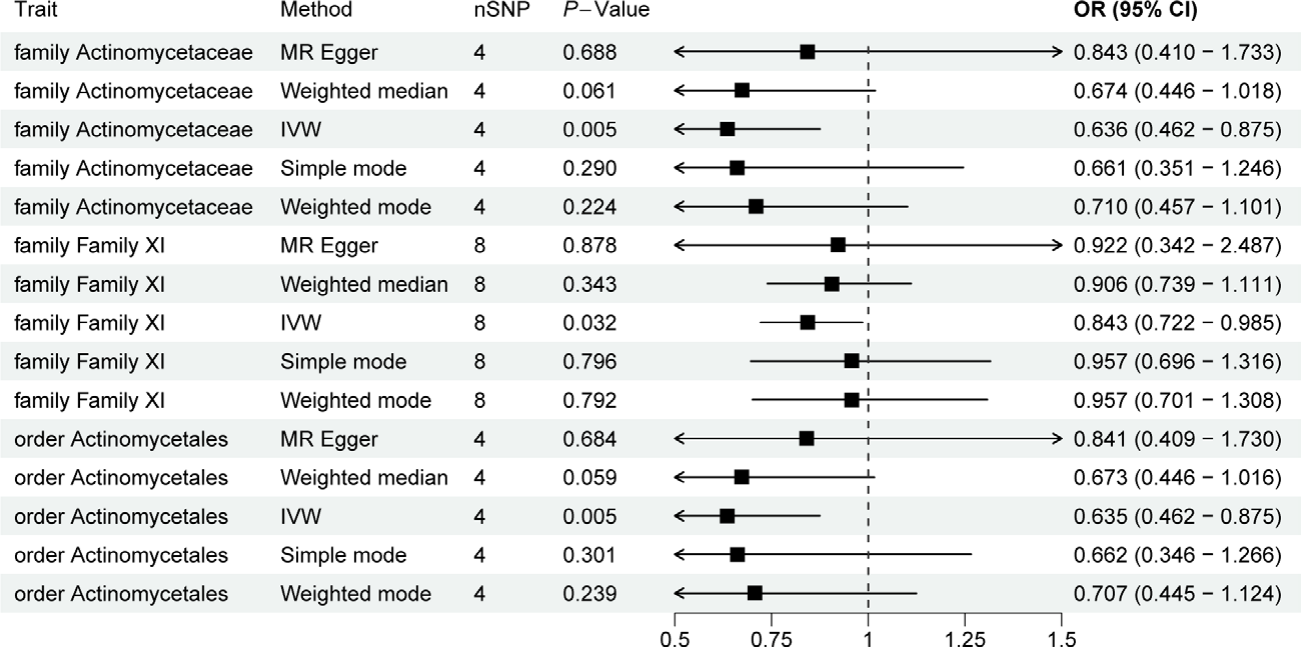
MR estimates for the association between GM and scoliosis. GM = gut microbiota, IVW = inverse variance weighted, MR = Mendelian randomization, nSNP = number of single nucleotide polymorphisms.

Using the IVW method, we identified a statistical correlation between 3 bacterial taxa and the risk of scoliosis (Fig. 2). The families Actinomycetaceae (OR = 0.636, 95% CI = 0.462–0.875, *P* = .005) and XI (OR = 0.843, 95% CI = 0.722−0.985, *P* = .032), and order Actinomycetales (OR = 0.635, 95% CI = 0.462−0.875, *P* = .005) were negatively associated with scoliosis, suggesting that the genetic prediction of these 3 gut microorganisms was associated with a reduced risk of scoliosis.

**Figure 3.**
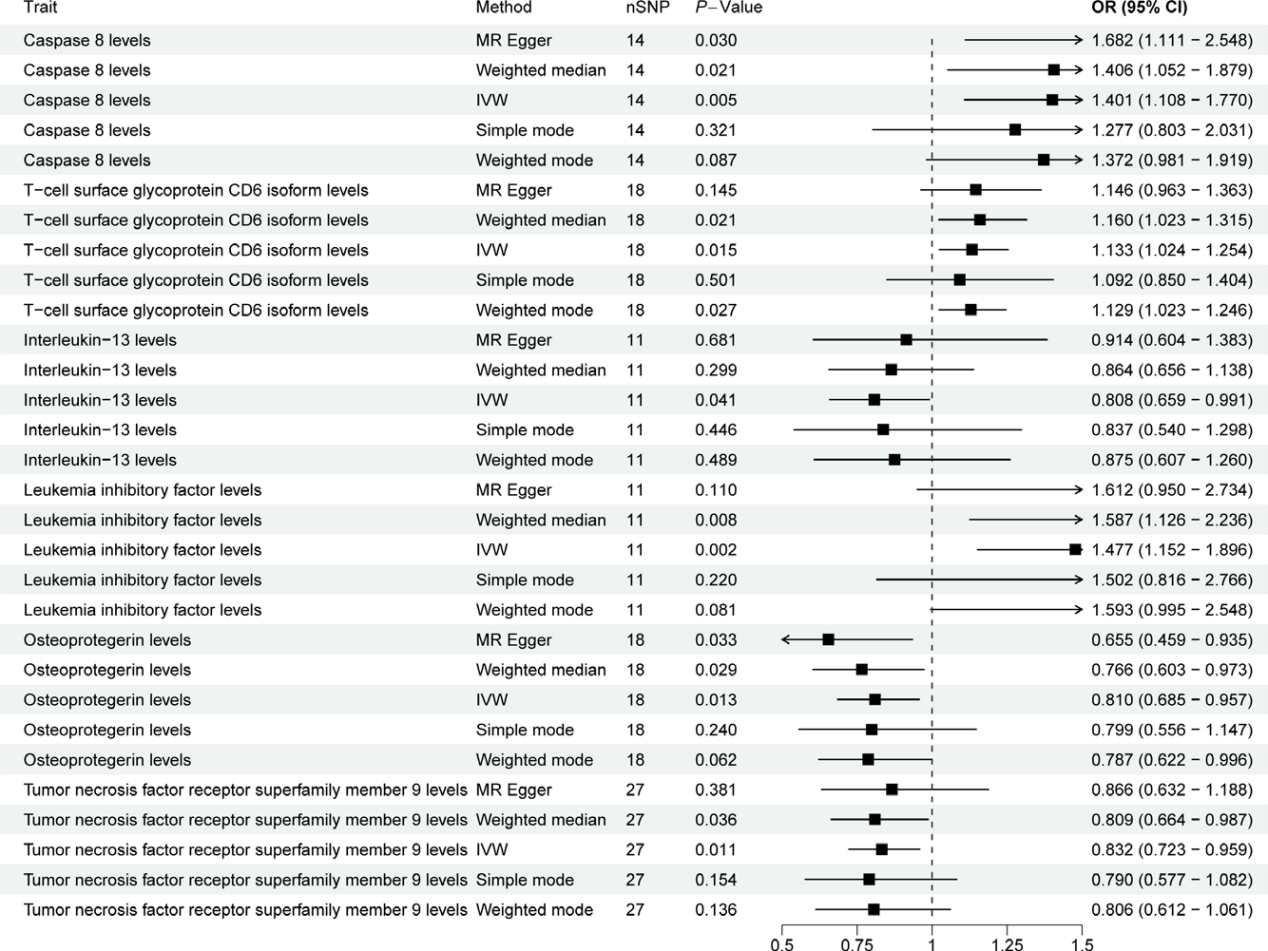
MR estimates for the association between IF and scoliosis. IF = inflammatory factor, IVW = inverse variance weighted, MR = Mendelian randomization, nSNP = number of single nucleotide polymorphisms.

Six IFs were significantly associated with scoliosis (Fig. 3). The levels of caspase 8 (OR = 1.401, 95% CI = 1.108−1.770, *P* = .005), T-cell surface glycoprotein CD6 isoform (OR = 1.133, 95% CI = 1.024–1.254, *P* = .015), and leukemia inhibitory factor (OR = 1.477, 95% CI = 1.152−1.896, *P* = .002) were positively associated with scoliosis, suggesting that the genetic prediction of these 3 IFs was associated with an increased risk of scoliosis. In contrast, levels of interleukin-13 (OR = 0.808, 95% CI = 0.659−0.991, *P* = .041), osteoprotegerin (OR = 0.810, 95% CI = 0.685–0.957, *P* = .013), and tumor necrosis factor receptor superfamily member 9 (OR = 0.832, 95% CI = 0.723–0.959, *P* = .011) were negatively correlated with scoliosis, suggesting that the genetic prediction of these 3 IFs was related to a reduced risk of scoliosis.

### 3.2. Causal effects of scoliosis on the GM and IFs

Our study showed that neither the GM, which was associated with scoliosis in forward MR, nor IFs, which were associated with scoliosis, were causally related in the reverse MR analysis. However, we found that scoliosis was significantly associated with the class Erysipelotrichia, family Bacteroidales S247 group, family Erysipelotrichaceae, family Peptococcaceae, genus *Alloprevotella*, genus *Coprococcus1*, genus *Eubacterium ventriosum* group, genus Family XIII AD3011 group, genus *Peptococcus*, genus *Roseburia*, genus *Ruminococcaceae* UCG004, genus *Ruminococcaceae* UCG005, genus *Ruminococcaceae* UCG010, and class Erysipelotrichales (Supplementary Table S1, Supplemental Digital Content, http://links.lww.com/MD/M878). In addition, our results also showed that scoliosis is significantly associated with the levels of T-cell surface glycoprotein CD6 isoform, hepatocyte growth factor, interleukin-18, and tumor necrosis factor ligand superfamily member 14 (Supplementary Table S2, Supplemental Digital Content, http://links.lww.com/MD/M879).

### 3.3. Sensitivity analyses

Both the IVW (*P* > .05) and MR-Egger (*P* > .05) methods showed no heterogeneity. Similarly, the MR-Egger regression intercept (*P* > .05) and MR-PRESSO global test (*P* > .05) demonstrated no pleiotropy (Table 1).

**Table 1:** Sensitivity analyze.

Exposure	Outcome	MR-Egger	IVW	MR-Egger intercept *P*	MR-PRESSO global test *P*
Family Actinomycetaceae	Scoliosis	0.341	0.399	.479	.550
Family Family XI		0.729	0.820	.864	.850
Order Actinomycetales		0.342	0.403	.482	.521
Caspase 8 levels		0.160	0.149	.317	.180
T-cell surface glycoprotein CD6 isoform levels		0.316	0.318	.879	.453
Interleukin-13 levels		0.659	0.702	.518	.737
Leukemia inhibitory factor levels		0.448	0.530	.722	.606
Osteoprotegerin levels		0.548	0.493	.203	.526
Tumor necrosis factor receptor superfamily member 9 levels		0.957	0.969	.785	.972

IVW = inverse variance weighted, MR-PRESSO = Mendelian randomization pleiotropy residual sum and outliers.

### 3.4. Mediation analysis

The findings of the MR analysis suggested a potential causal link between GM associated with scoliosis and IFs associated with the condition (Supplementary Table S3, Supplemental Digital Content, http://links.lww.com/MD/M880). IFs appear to play a role in the pathway through which GM mediates scoliosis. However, using MVMR and correcting for reciprocal effects, the causal effects between GM and IFs and scoliosis were not significant (Supplementary Table S4, Supplemental Digital Content, http://links.lww.com/MD/M881). This suggests that a causal relationship between the IFs that mediate GM and scoliosis has not yet been established.

## 4. Discussion

Our results identified 3 negative correlations between GM and scoliosis. Furthermore, we found 3 positive and 3 negative correlations between IFs and scoliosis. However, no evidence suggests that IFs act as mediators in the pathway from the GM to scoliosis.

The GM, as a core part of the internal environment, forms a symbiotic relationship with its host and has a significant impact on maintaining host health.^[33]^ The GM consists of approximately 3000 to 5000 microorganisms,^[34]^ which are extremely complex in structure and function, maintaining a dynamic balance in a healthy physiological state. The GM is recognized as an important organ for regulating gene expression associated with the immune system, food digestion, and energy metabolism.

The “gut-bone” axis has recently received much attention.^[35]^ It has been observed that the GM is associated with, or even causally related to, various diseases, including obesity, metabolic syndrome, malnutrition, rheumatoid arthritis, and spondyloarthropathies,^[15]^ and contributes to the development of bone loss and secondary osteoporosis.^[36]^ Bone homeostasis has also been demonstrated to be affected by the GM composition or production.^[37]^ Yan and Charles^[38]^ found that the GM regulates and stimulates host insulin-like growth factor I (IGF-I), a key factor in bone growth, through dynamic interactions. *Bifidobacteria* and *Lactobacillus* exhibit anti-inflammatory properties that can enhance vitamin D absorption and inhibit osteoclast development, thereby mitigating bone loss following ovariectomy in mice.^[39,40]^ Conversely, analysis of 16S rRNA gene sequencing data indicated an elevation in the abundance of bacteria under the phylum Proteobacteria (e.g., *Pseudomonas* and *Enterobacter*) in women with postmenopausal osteopenia.^[41]^ Both probiotics and prebiotics have also been shown to have a positive impact on reversing bone loss in vivo, thereby broadening therapeutic options for treating osteoporosis.^[37]^ These evidence suggests that GM dysbiosis plays a role in musculoskeletal health.

Given the varied forms of scoliosis and the intricate nature of the GM, summarizing the impact of GM on scoliosis in observational research is challenging. This study employed an MR approach to examine the potential causal link between GM and scoliosis. Our analysis focused on the abundance of 211 prevalent GM taxa and their associations with scoliosis. These findings indicate that certain taxa, such as the family Actinomycetaceae, family XI, and order Actinomycetales, are protective factors against scoliosis. However, in an MR study, a positive association was observed between the family Actinomycetaceae and lumbar spine bone mineral density.^[42]^ Moreover, a study conducted in 2016 showed correlations between low bone density and an increased likelihood of developing a curved spine in adolescents with scoliosis. Furthermore, a 2019 study cited previous research suggesting that individuals with osteoporosis have a higher propensity to develop age-related scoliosis.^[43]^ The diminished bone mineral density characteristic of osteoporosis compromises the structural integrity of the vertebrae in the spinal column, thereby increasing the susceptibility to vertebral compression fractures resulting from mechanical stress. This pathological condition may predispose older individuals to scoliosis.^[44]^

The FinnGen Consortium R10 data source indicated a peak in the onset of scoliosis among adolescents under 20 years of age, suggesting that adolescent idiopathic scoliosis is the most prevalent spinal deformity. Discrepancies in the species studied and variations in the abundance of Actinomycetaceae strains may have contributed to these conflicting findings. As identified in this study, the relationship between GM and scoliosis remains unclear. Additional research is essential to elucidate the mechanisms underlying the beneficial and detrimental effects of different GM taxa on scoliosis.

In the present study, the relative abundances of various GM taxa were used to determine their relevance to scoliosis. However, the specific mechanism by which GM influences scoliosis remains unclear. We hypothesized that IFs mediate the relationship between GM and scoliosis. We investigated the association between 91 common IFs and scoliosis. We found that levels of interleukin-13, osteoprotegerin, and tumor necrosis factor receptor superfamily member 9 were protective factors against scoliosis. Moreover, the levels of caspase 8, T-cell surface glycoprotein CD6 isoform, and leukemia inhibitory factor significantly increased the risk of scoliosis. Previous research showed that caspase 8 promoted osteoblast proliferation.^[45]^ Sakamoto et al^[46]^ found that osteoblasts were more sensitive to the inhibitory effects of glucose-dependent insulinotropic polypeptide receptor (GiPCR) signaling and that inhibiting GiPCR signaling is important in driving the development of scoliosis. This seems to be the reason why caspase 8 increases the risk of scoliosis.

Scoliosis itself may influence variations in the GM and inflammatory factors. Therefore, we evaluated the causal relationship between scoliosis, the GM, and IFs. The results indicated that scoliosis was causally related to 14 GM taxa, including the class Erysipelotrichia, family Bacteroidales S247 group, family Erysipelotrichaceae, family Peptococcaceae, genus *Alloprevotella*, genus *Coprococcus1*, genus *Eubacterium ventriosum* group, genus Family XIII AD3011 group, genus *Peptococcus*, genus *Roseburia*, genus *Ruminococcaceae* UCG004, genus *Ruminococcaceae* UCG005, genus *Ruminococcaceae* UCG010, and order Erysipelotrichales. In addition, scoliosis was causally related to 4 common IFs, including T-cell surface glycoprotein CD6 isoform, hepatocyte growth factor, interleukin-18, and tumor necrosis factor ligand superfamily members.

This study represents the first MR analysis to assess the relationship between human GM and scoliosis, considering the potential mediating role of IFs. Our findings provide a valuable reference for identifying biomarkers and therapeutic targets to manage scoliosis and address clinical challenges. Moreover, our study provides additional insights and avenues for understanding the mechanisms underlying scoliosis development.

However, despite offering numerous promising avenues for scoliosis prevention and intervention, our study had several limitations. First, our study utilized pooled data instead of raw data. Consequently, we did not perform subgroup analyses, such as distinguishing between types of scoliosis. Second, most participants in the GWAS data source were of European origin, thus limiting the generalizability of the findings to non-European populations. Third, while we investigated the mediating role of IFs between different gut flora abundances and scoliosis, the mechanisms through which gut flora influences the onset of scoliosis require further investigation, as IFs do not serve as mediators.

## 5. Conclusion

Our study investigated bidirectional causal relationships between the GM, IFs, and scoliosis. Three negative causal correlations were observed between the genetic predisposition to GM and scoliosis. Positive and negative correlations were found between IFs and scoliosis, with 3 positive and 3 negative correlations identified. In our analysis, IFs did not act as mediators in the pathway from the GM to scoliosis.

## Acknowledgments

The authors appreciate the original study investigators for sharing their valuable information.

## Author contributions

**Writing – original draft:** Xiaojiang Zhao, Changqing Li.

**Writing – review & editing:** Xiaojiang Zhao, Lei Zhang, Changqing Li.

**Data curation:** Jingjing Liu, Chao Ma.

**Visualization:** Yanan Liu, Hebao Wen.

**Funding acquisition:** Changqing Li.

## Supplementary Material








