# Supplementary material for: Gut microbiota, inflammatory factors, and scoliosis: A Mendelian randomization study
Source: Medicine (Baltimore). 2024 Jun 14;103(24):e38561. doi: 10.1097/MD.0000000000038561 (PMC11175948; doi:10.1097/MD.0000000000038561)
Supplement: Supplementary file 1 [file medi-103-e38561-s001.doc]

| **Supplementary Table** S1**. The causal effects of scoliosis on gut microbiota** | | | | | |
| --- | --- | --- | --- | --- | --- |
| **Exposure** | **Outcome** | **nsnp** | **Methods** | **OR(95%CI)** | **p** |
| scoliosis | class Erysipelotrichia | 15 | MR Egger | 0.93(0.72 to 1.19) | 0.57 |
|  |  |  | Weighted median | 0.97(0.92 to 1.03) | 0.36 |
|  |  |  | Inverse variance weighted | 0.95(0.91 to 1.00) | 0.03 |
|  |  |  | Simple mode | 0.98(0.89 to 1.08) | 0.65 |
|  |  |  | Weighted mode | 0.98(0.89 to 1.07) | 0.62 |
| scoliosis | family Bacteroidales S247group | 15 | MR Egger | 1.57(1.09 to 2.26) | 0.03 |
|  |  |  | Weighted median | 1.07(0.98 to 1.17) | 0.13 |
|  |  |  | Inverse variance weighted | 1.08(1.01 to 1.15) | 0.02 |
|  |  |  | Simple mode | 1.04(0.88 to 1.23) | 0.64 |
|  |  |  | Weighted mode | 1.05(0.90 to 1.22) | 0.54 |
| scoliosis | family Erysipelotrichaceae | 15 | MR Egger | 0.93(0.72 to 1.19) | 0.57 |
|  |  |  | Weighted median | 0.97(0.92 to 1.03) | 0.35 |
|  |  |  | Inverse variance weighted | 0.95(0.91 to 1.00) | 0.03 |
|  |  |  | Simple mode | 0.98(0.89 to 1.08) | 0.66 |
|  |  |  | Weighted mode | 0.98(0.89 to 1.07) | 0.60 |
| scoliosis | family Peptococcaceae | 15 | MR Egger | 1.05(0.77 to 1.43) | 0.76 |
|  |  |  | Weighted median | 0.93(0.87 to 1.01) | 0.07 |
|  |  |  | Inverse variance weighted | 0.94(0.89 to 1.00) | 0.04 |
|  |  |  | Simple mode | 0.92(0.81 to 1.04) | 0.20 |
|  |  |  | Weighted mode | 0.91(0.81 to 1.03) | 0.17 |
| scoliosis | genus Alloprevotella | 6 | MR Egger | 0.35(0.03 to 3.97) | 0.44 |
|  |  |  | Weighted median | 0.84(0.69 to 1.02) | 0.08 |
|  |  |  | Inverse variance weighted | 0.84(0.72 to 0.99) | 0.04 |
|  |  |  | Simple mode | 0.91(0.69 to 1.20) | 0.53 |
|  |  |  | Weighted mode | 0.79(0.60 to 1.03) | 0.14 |
| scoliosis | genus Coprococcus1 | 15 | MR Egger | 0.89(0.69 to 1.15) | 0.39 |
|  |  |  | Weighted median | 0.95(0.89 to 1.01) | 0.07 |
|  |  |  | Inverse variance weighted | 0.94(0.90 to 0.99) | 0.01 |
|  |  |  | Simple mode | 0.98(0.89 to 1.09) | 0.77 |
|  |  |  | Weighted mode | 0.91(0.82 to 1.01) | 0.09 |
| scoliosis | genus Eubacterium ventriosum group | 15 | MR Egger | 0.92(0.71 to 1.20) | 0.56 |
|  |  |  | Weighted median | 0.94(0.89 to 1.01) | 0.07 |
|  |  |  | Inverse variance weighted | 0.94(0.89 to 0.98) | 0.01 |
|  |  |  | Simple mode | 0.95(0.85 to 1.06) | 0.34 |
|  |  |  | Weighted mode | 0.95(0.85 to 1.06) | 0.39 |
| scoliosis | genus Family XIII AD3011 group | 15 | MR Egger | 1.04(0.79 to 1.36) | 0.81 |
|  |  |  | Weighted median | 0.96(0.90 to 1.03) | 0.25 |
|  |  |  | Inverse variance weighted | 0.95(0.90 to 1.00) | 0.04 |
|  |  |  | Simple mode | 0.97(0.87 to 1.07) | 0.56 |
|  |  |  | Weighted mode | 0.97(0.88 to 1.07) | 0.55 |
| **Exposure** | **Outcome** | **nsnp** | **Methods** | **OR(95%CI)** | **p** |
| scoliosis | genus Peptococcus | 15 | MR Egger | 0.79(0.52 to 1.20) | 0.30 |
|  |  |  | Weighted median | 0.91(0.83 to 1.01) | 0.09 |
|  |  |  | Inverse variance weighted | 0.92(0.85 to 0.99) | 0.03 |
|  |  |  | Simple mode | 0.92(0.78 to 1.09) | 0.35 |
|  |  |  | Weighted mode | 0.94(0.79 to 1.11) | 0.47 |
| scoliosis | genus Roseburia | 15 | MR Egger | 1.30(1.02 to 1.66) | 0.06 |
|  |  |  | Weighted median | 1.06(0.99 to 1.12) | 0.08 |
|  |  |  | Inverse variance weighted | 1.06(1.01 to 1.11) | 0.02 |
|  |  |  | Simple mode | 1.04(0.93 to 1.16) | 0.53 |
|  |  |  | Weighted mode | 1.05(0.94 to 1.16) | 0.40 |
| scoliosis | genus Ruminococcaceae UCG004 | 15 | MR Egger | 1.27(0.91 to 1.76) | 0.18 |
|  |  |  | Weighted median | 1.06(0.97 to 1.15) | 0.21 |
|  |  |  | Inverse variance weighted | 1.07(1.01 to 1.14) | 0.02 |
|  |  |  | Simple mode | 1.02(0.89 to 1.18) | 0.74 |
|  |  |  | Weighted mode | 1.05(0.91 to 1.21) | 0.54 |
| scoliosis | genus Ruminococcaceae UCG005 | 15 | MR Egger | 0.98(0.76 to 1.27) | 0.90 |
|  |  |  | Weighted median | 0.95(0.89 to 1.02) | 0.14 |
|  |  |  | Inverse variance weighted | 0.95(0.91 to 1.00) | 0.04 |
|  |  |  | Simple mode | 0.95(0.85 to 1.06) | 0.37 |
|  |  |  | Weighted mode | 0.95(0.86 to 1.05) | 0.35 |
| scoliosis | genus Ruminococcaceae UCG010 | 15 | MR Egger | 1.16(0.87 to 1.55) | 0.32 |
|  |  |  | Weighted median | 0.92(0.86 to 0.98) | 0.02 |
|  |  |  | Inverse variance weighted | 0.94(0.89 to 0.99) | 0.02 |
|  |  |  | Simple mode | 0.91(0.81 to 1.03) | 0.16 |
|  |  |  | Weighted mode | 0.91(0.81 to 1.03) | 0.17 |
| scoliosis | order Erysipelotrichales | 15 | MR Egger | 0.93(0.72 to 1.19) | 0.57 |
|  |  |  | Weighted median | 0.97(0.91 to 1.03) | 0.37 |
|  |  |  | Inverse variance weighted | 0.95(0.91 to 1.00) | 0.03 |
|  |  |  | Simple mode | 0.98(0.89 to 1.08) | 0.65 |
|  |  |  | Weighted mode | 0.98(0.89 to 1.07) | 0.61 |
