# Supplementary material for: Gut microbiota, inflammatory factors, and scoliosis: A Mendelian randomization study
Source: Medicine (Baltimore). 2024 Jun 14;103(24):e38561. doi: 10.1097/MD.0000000000038561 (PMC11175948; doi:10.1097/MD.0000000000038561)
Supplement: Supplementary file 2 [file medi-103-e38561-s002.doc]

| **Supplementary Table** S2**. The causal effects of scoliosis on Inflammatory factor** | | | | | | | |
| --- | --- | --- | --- | --- | --- | --- | --- |
| **Exposure** | **Outcome** | **nsnp** | **Methods** | **OR** | **or_lci95** | ***or_uci95*** | ***P*** |
| DLB | T-cell surface glycoprotein CD6 isoform levels | 27 | MR Egger | 0.99 | 0.93 | 1.05 | 0.69 |
|  |  |  | Weighted median | 1.03 | 0.97 | 1.08 | 0.33 |
|  |  |  | Inverse variance weighted | 1.04 | 1.00 | 1.07 | 0.03 |
|  |  |  | Simple mode | 1.01 | 0.93 | 1.10 | 0.79 |
|  |  |  | Weighted mode | 1.02 | 0.96 | 1.08 | 0.52 |
| DLB | Hepatocyte growth factor levels | 27 | MR Egger | 0.91 | 0.86 | 0.97 | 0.01 |
|  |  |  | Weighted median | 0.95 | 0.91 | 1.00 | 0.06 |
|  |  |  | Inverse variance weighted | 0.97 | 0.93 | 1.00 | 0.03 |
|  |  |  | Simple mode | 0.97 | 0.88 | 1.07 | 0.58 |
|  |  |  | Weighted mode | 0.93 | 0.86 | 0.99 | 0.04 |
| DLB | Interleukin-18 levels | 27 | MR Egger | 0.90 | 0.84 | 0.96 | 0.00 |
|  |  |  | Weighted median | 0.95 | 0.90 | 0.99 | 0.03 |
|  |  |  | Inverse variance weighted | 0.96 | 0.92 | 0.99 | 0.03 |
|  |  |  | Simple mode | 0.96 | 0.88 | 1.05 | 0.39 |
|  |  |  | Weighted mode | 0.94 | 0.88 | 1.00 | 0.06 |
| DLB | Tumor necrosis factor ligand superfamily member 14 | 27 | MR Egger | 0.92 | 0.86 | 0.99 | 0.04 |
|  |  |  | Weighted median | 0.94 | 0.89 | 1.00 | 0.05 |
|  |  |  | Inverse variance weighted | 0.95 | 0.92 | 0.99 | 0.01 |
|  |  |  | Simple mode | 0.94 | 0.85 | 1.03 | 0.21 |
|  |  |  | Weighted mode | 0.94 | 0.88 | 1.01 | 0.13 |
