# Supplementary material for: Gut microbiota, inflammatory factors, and scoliosis: A Mendelian randomization study
Source: Medicine (Baltimore). 2024 Jun 14;103(24):e38561. doi: 10.1097/MD.0000000000038561 (PMC11175948; doi:10.1097/MD.0000000000038561)
Supplement: Supplementary file 3 [file medi-103-e38561-s003.doc]

| Supplementary Table S3. Causal effects of gut microbiota associated with scoliosis on inflammatory factor associated with scoliosis | | | | | |
| --- | --- | --- | --- | --- | --- |
| **Exposure** | **Outcome** | **nsnp** | **Methods** | **OR(95%CI)** | **p** |
| family Actinomycetaceae | Leukemia inhibitory factor levels | 5 | MR Egger | 0.73(0.49 to 1.10) | 0.23 |
|  |  |  | Weighted median | 0.87(0.71 to 1.07) | 0.20 |
|  |  |  | Inverse variance weighted | 0.85(0.73 to 0.99) | 0.04 |
|  |  |  | Simple mode | 0.88(0.67 to 1.17) | 0.43 |
|  |  |  | Weighted mode | 0.87(0.67 to 1.14) | 0.37 |
| order Actinomycetales | Leukemia inhibitory factor levels | 5 | MR Egger | 0.73(0.49 to 1.10) | 0.23 |
|  |  |  | Weighted median | 0.87(0.71 to 1.07) | 0.20 |
|  |  |  | Inverse variance weighted | 0.85(0.73 to 0.99) | 0.04 |
|  |  |  | Simple mode | 0.88(0.68 to 1.15) | 0.41 |
|  |  |  | Weighted mode | 0.87(0.68 to 1.12) | 0.34 |
