# Supplementary material for: Gut microbiota, inflammatory factors, and scoliosis: A Mendelian randomization study
Source: Medicine (Baltimore). 2024 Jun 14;103(24):e38561. doi: 10.1097/MD.0000000000038561 (PMC11175948; doi:10.1097/MD.0000000000038561)
Supplement: Supplementary file 4 [file medi-103-e38561-s004.doc]

| SupplementaryTable S4. Causal relationship between gut microbiota and inflammatory factors and scoliosis by multivariate Mendelian randomization (MVMR) | | | | | |
| --- | --- | --- | --- | --- | --- |
| **Exposure** | **Outcome** | **nsnp** | **beta** | **se** | **p** |
| amily Actinomycetaceae | scoliosis | 4 | -0.39 | 0.18 | 0.03 |
| Leukemia inhibitory factor levels | scoliosis | 17 | 0.15 | 0.16 | 0.32 |
| order Actinomycetales | scoliosis | 4 | -0.39 | 0.18 | 0.03 |
| Leukemia inhibitory factor levels | scoliosis | 17 | 0.15 | 0.16 | 0.32 |
